# Columbianetin alleviates lipopolysaccharides (LPS)-induced inflammation and apoptosis in chondrocyte through activation of autophagy by inhibiting serum and glucocorticoid-induced protein kinase 1 (SGK1) expression

**DOI:** 10.1080/21655979.2022.2032970

**Published:** 2022-02-07

**Authors:** Wei Chen, Haotian Zheng, Xuan Zhang, Yude Xu, Zhibin Fu, Xing Ji, Changhao Wei, Guoyao An, Mingyuan Tan, Mingwang Zhou

**Affiliations:** a Department of Orthopaedics, Traditional Chinese Medical Hospital of Gansu Province, Lanzhou, Gansu, China; b Clinical College of Chinese Medicine, Gansu University of Chinese Medicine, Lanzhou, Gansu, China; c Department of Oncology, Traditional Chinese Medical Hospital of Gansu Province, Lanzhou, Gansu, China

**Keywords:** Columbianetin, SGK1, autophagy, osteoarthritis, inflammation

## Abstract

Osteoarthritis (OA) is a degenerative disease of articular cartilage involving the entire joint tissue. Columbianetin (CBT) is a major active compound of radix angelicae pubescentis, which is used in the treatment of OA. This paper attempts to explore the role of CBT in OA. Lipopolysaccharides (LPS) was used to induce mouse chondrocytes ATDC5. The effect of CBT on cell viability in ATDC5 cells with or without LPS induction was determined by CCK-8 and LDH kits. The inflammatory response was evaluated using ELISA kits. Apoptosis in LPS-induced ATDC5 cells were examined by TUNEL staining. The expression of apoptosis and autophagy-related proteins was tested with Western blot. The relationship between CBT and serum and glucocorticoid-induced protein kinase 1 (SGK1) was examined by RT-qPCR, Western blot, and molecular docking. After SGK1 overexpression or addition of the autophagy inhibitor 3-methyladenine (3 MA), the above experiments were done again. Results revealed that CBT increased LPS-induced decrease in ATDC5 cell viability. CBT inhibited inflammation triggered by LPS, evidenced by reduced levels of TNF-α, IL-6 and IL-1β. Cell apoptosis was attenuated following CBT adding in ATDC5 cells exposed to LPS, accompanied by upregulated Bcl-2 expression and downregulated Bax and cleaved caspase 3 expression. In addition, CBT elevated Beclin1 and LC3II/LC3I expression but decreased p62 expression. Additionally, CBT inhibited SGK1 expression. However, SGK1 overexpression or 3 MA reversed the effects of CBT on LPS-induced loss of ATDC5 cell viability, inflammation, apoptosis and autophagy. Collectively, CBT could improve OA through the activation of chondrocyte autophagy by suppressing SGK1 expression.

## Graphical abstract

**Figure:**
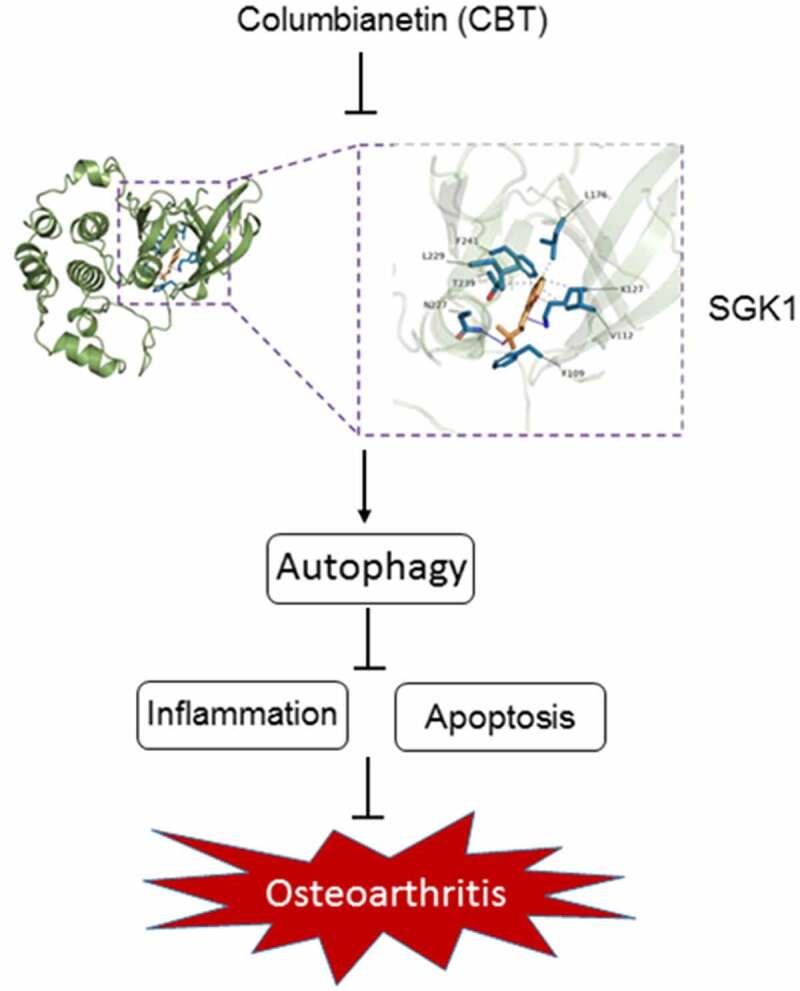

## Introduction

Osteoarthritis (OA) is a degenerative joint disease that seriously affects the health of the elderly, and the morbidity and mortality are increasing worldwide [1]. The main clinical symptoms of OA include chronic pain, joint instability, stiffness, joint deformity and radiographic joint space narrowing [2,3]. Commonly used treatments are low-intensity aerobic exercise, weight loss, acupuncture, glucosamine, chondroitin sulfate, and surgery [4]. However, most of the treatments can only reduce the pain and stiffness of the joints, and the treatment effect is still not obvious.

Chondrocytes are mainly found in adult articular cartilage and play an important role in maintaining the matrix composition [5]. However, a previous study has argued that activation of external stress and inflammation-inducing signals may lead to phenotypic transformation of chondrocytes, apoptosis and aberrant expression of inflammation-related genes [6]. Accordingly, inflammation and chondrocyte apoptosis are thought to be the pathological causes of OA joint degeneration. And it is widely believed that activation of chondrocyte autophagy can effectively improve OA [7–9]. Therefore, activating chondrocyte autophagy and maintaining normal chondrocyte function are important ways to improve OA.

Columbianetin (CBT) is one of the major ingredients separated from the root of radix angelicae pubescentis, with multiple bioactivities, such as antioxidative, antiproliferation, anti-inflammatory, and anti-nitric oxide production activities [10,11]. CBT is reported to exert anti-inflammation effect in human peripheral blood mononuclear cells and human mast cells [10]. However, its role in OA has not been reported. Additionally, serum and glucocorticoid inducible kinase-1 (SGK1) is a widely expressed serine/threonine kinase located downstream of the PI3K signaling pathway [12]. Overexpression of SGK1 is associated with the development of various diseases, including hypertension, obesity, fibrotic diseases, vascular calcification and tumor growth [13]. While the inhibition of SGK1 not only induces cellular autophagy and apoptosis and delays prostate cancer progression, but also activates cell autophagy and reverses IL-1β-induced anabolic and catabolic imbalance in chondrocytes [14,15]. Some studies have pointed out that SGK1 expression was found to be upregulated in OA and inhibition of SGK1 expression was effective in improving OA [16,17].

The aim of the present study was to investigate the role and possible mechanism of CBT in OA. Lipopolysaccharides (LPS)-induced mouse chondroprogenitor cell line (ATDC5) was used to simulate OA model in vitro. The effects of CBT on LPS-triggered inflammation and apoptosis of ATDC5 cells were explored. Additionally, the latent regulatory mechanism of CBT related to SGK1 was explored. Our findings might provide a potential therapeutic agent for OA.

## Materials and methods

### Cell culture and treatment

The mouse chondroprogenitor cells ATDC5 (American Type Culture Collection; Manassas, VA, USA) were plated in the medium filling with a mixture of Dulbecco’s Modified Eagle Medium (DMEM) and F12 Ham containing 5% fetal bovine serum (FBS; HyClone, Logan, UT, USA) in 37°C and 5% CO_2_. To construct an *in vitro* osteoarthritic chondrocyte model, ATDC5 cells were induced with 5 μg/mL of LPS for 24 h according to the previous study [18]. To observe the effect of CBT on ATDC5 cells, CBT at a concentration of 10, 20, 40 μg/ml was adopted to treat ATDC5 cells for another 24 h. The autophagy inhibitor 3-methyladenine (3 MA; 10 mM) was adopted to pretreat ATDC5 cells for 24 h [19].

### Cell transfection

To enable ATDC5 cells to stably present SGK1 overexpression, the overexpression plasmid of SGK1 (Oe-SGK1) and the empty plasmid (Oe-NC) were designed and provided by Gene Pharma Company (Shanghai, China). Later, these vectors were then transfected into ATDC5 cells with the application of Lipofectamine 2000 (Invitrogen, Carlsbad, CA, USA). The successful transfection was evaluated via reverse transcription-quantitative PCR (RT-qPCR) and Western blot assays at 48 h after transfection.

### Cell viability assay

Cell viability detection was carried out by means of cell counting kit (CCK)-8 method. ATDC5 cells were plated into 96-well plates at a density of 5 × 10^3^ cells/well. Before and after LPS treatment, ATDC5 cells were cultured with CCK-8 solution (Shanghai Yeasen Biotechnology; Shanghai, China) in the condition of 95% air, 5% CO_2_ and 37°C for 4 h. The absorbance at 450 nm was read with the aid of a Microplate reader (Flash Spectrum Biotechnology, Shanghai, China).

### Lactate dehydrogenase (LDH) release

LDH activity was assayed with the use of LDH assay kit (Nanjing Jiancheng Bioengineering Institute; Nanjing, China). Following the recommendations of manufacturer, ATDC5 cells were seeded into 96-well plates and incubated with LDH assay reagent for 30 min. Finally, the content of LDH was tested at 490 nm with a spectrophotometer (Mettler Toledo, Shanghai, China).

### RT-qPCR

Total RNA from treated ATDC5 cells was placed in Trizol reagent (Ambion, Austin, TX, USA), followed by a reverse transcription of 20 μg total RNA with a Taqman MicroRNA Reverse Transcription Kit (Invitrogen) to obtain complementary DNA (cDNA). The detection of SGK1 mRNA expression was undertaken with the help of ABI 7500 system (Applied Biosystems, Carlsbad, CA, USA) employing a QuantiNova SYBR Green PCR Kit (Qiagen, Germantown, MD, USA). The conditions required for this reaction were 95°C for 2 min, 95°C for 5 s, 60°C for 10s, 40 cycles in total. The primer sequence was shown below: SGK1: forward, 5’-AGGATGGGTCTGAACGACTTT-3’, reverse, 5’-GCCCTTTCCGATCACTTTCAAG-3’; Glyceraldehyde-phosphate dehydrogenase (GAPDH): forward, 5’-CTACCCCCAATGTGTCCGTC-3’, reverse, 5’-GGCCTCTCTTGCTCAGTGTC-3’. Quantification was undertaken in accordance with a comparative Ct (2^−ΔΔCT^) method [20].

### Western blot

The protein from ATDC5 cells was extracted through radioimmunoprecipitation (RIPA) lysis buffer (Beyotime, Shanghai, China) containing protease inhibitors (Roche, Basel, Switzerland). The quantification of extracted proteins was carried out utilizing bicinchoninic acid assay (BCA) Protein Assay Kit (Pierce, Appleton, WI, USA). Subsequently, after being separated by electrophoresis with 10% sodium dodecyl sulfate-polyacrylamide gel electrophoresis (SDS-PAGE) gels, the proteins were transferred to polyvinylidene difluoride (PVDF) membranes. After blocking with 5% skimmed milk, theses membranes were incubated with the primary antibodies specific against B-cell lymphoma 2 (Bcl-2; Abcam, ab32124, 1:1000), Bcl2-associated X (Bax; Abcam, ab32503, 1:1000), cleaved caspase 3 (Abcam, ab32042, 1:1000), caspase 3 (Abcam, ab32351, 1:1000), Beclin1 (Abcam, ab207612, 1:2000), LC3II/LC3I (Abcam, ab192890, 1:2000), p62 (Abcam, ab140651, 1:10000), SGK1 (Abcam, ab32374, 1:500) overnight at 4℃. The secondary antibody carrying with horseradish peroxidase (HRP)-conjugates was added for 1 h of incubation at room temperature. After washing with TBST three times, the protein blots were detected using an enhanced chemiluminescent (ECL) luminescence reagent (Sangon Biotech, Shanghai, China). The gray values were normalized to the intensity of the corresponding bands for GAPDH. Quantitative analysis of proteins was performed employing ImageJ software (National Institutes of Health, Bethesda, MA, USA).

### Cell apoptosis assessment

The evaluation of apoptotic cells was conducted with the adoption of terminal-deoxynucleoitidyl transferase mediated nick end labeling (TUNEL) method according to the instructions of a TUNEL Apoptosis Detection kit (Beyotime, Shanghai, China). Briefly, ATDC5 cells were washed three times with phosphate buffer saline (PBS) for 3 min each, and fixed with pre-cooled ethanol for 5 min. Next, these cells were exposed to TdT enzyme for 30 min at 37°C, followed by an incubation with 4’,6-diamidino-2-phenylindole (DAPI) for cell nuclear staining. Finally, the staining ATDC5 cells were photographed employing a Zeiss LSM-510 Meta confocal microscope (Zeiss, Beijing, China).

### Enzyme linked immunosorbent assay (ELISA)

After ATDC5 cells were treated with LPS and/or CBT as well as Oe-SGK1 or 3 MA, the supernatant was collected for the detection of inflammatory cytokines levels. The concentration of inflammatory cytokines tumor necrosis factor (TNF)-α, interleukin (IL)-6 and IL-1β was determined by means of corresponding ELISA kits (Shanghai XiTang Biotechnology; Shanghai, China) in keeping with the manufacturer’s standard procedures. The absorbance was read by a microplate reader at 450 nm.

### Molecular docking

The interaction of CBT and SGK1 was predicted by molecular docking method. The structures of CBT and SGK1 were obtained from the PDB database (https://www.rcsb.org/) and constructed by the Molecular Operating Environment software. Subsequently, the 3D structures of CBT and SGK1 were matched. The 32-bit command program CMD.exe was used to calculate the affinity between CBT and SGK1.

### Statistical analysis

All of the experiments were repeated three times (n = 3) and data were shown as mean ± standard deviation (SD). Statistical analysis was carried out by GraphPad Prism 8.0 software (GraphPad Software Inc., La Jolla, CA, USA). The comparison was determined by Student's t-test and one-way analysis of variance (ANOVA). A P value <0.05 was a statistically significant result.

## Results

### CBT increases LPS-induced ATDC5 cell viability

**Figure 1.**
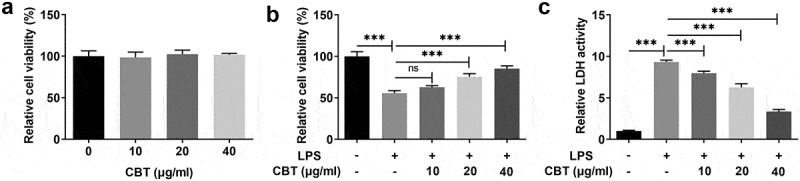
CBT increases LPS-induced ATDC5 cell viability.

CBT has been reported to possess multiple beneficial bioactivities, such as antioxidative, anti-inflammatory and anti-nitric oxide production activities [10,11]. To assess the influence of CBT on cell viability of LPS-induced ATDC5 cells, we first conducted the CCK-8 experiment. As shown in Figure 1(a), the viability of ATDC5 cells treated with CBT at concentrations of 10, 20 and 40 μg/ml showed no obvious changes compared with the control group. It was found in Figure 1(b) that the viability of ATDC5 cells decreased by about 45% in the LPS group (vs Control) and increased in a concentration-dependent manner after treatment with different concentrations of CBT. Moreover, LPS induced a high LDH activity (vs Control), but treatment of CBT concentration-dependently reduced the LDH activity (Figure 1(c)). These results support the notion that CBT prevents LPS-induced loss of ATDC5 cell viability.

(a) Cell viability of ATDC5 cells treated with CBT (10, 20 and 40 μg/ml) was assessed utilizing CCK-8. (b) Cell viability of LPS-induced ATDC5 cells treated with CBT (10, 20 and 40 μg/ml) was tested adopting CCK-8. (c) LDH release level in LPS-induced ATDC5 cells treated with CBT (10, 20 and 40 μg/ml) was examined with the use of LDH kit. ***P < 0.001.

### CBT ameliorates LPS-induced inflammatory response and apoptosis in ATDC5 cells

**Figure 2.**
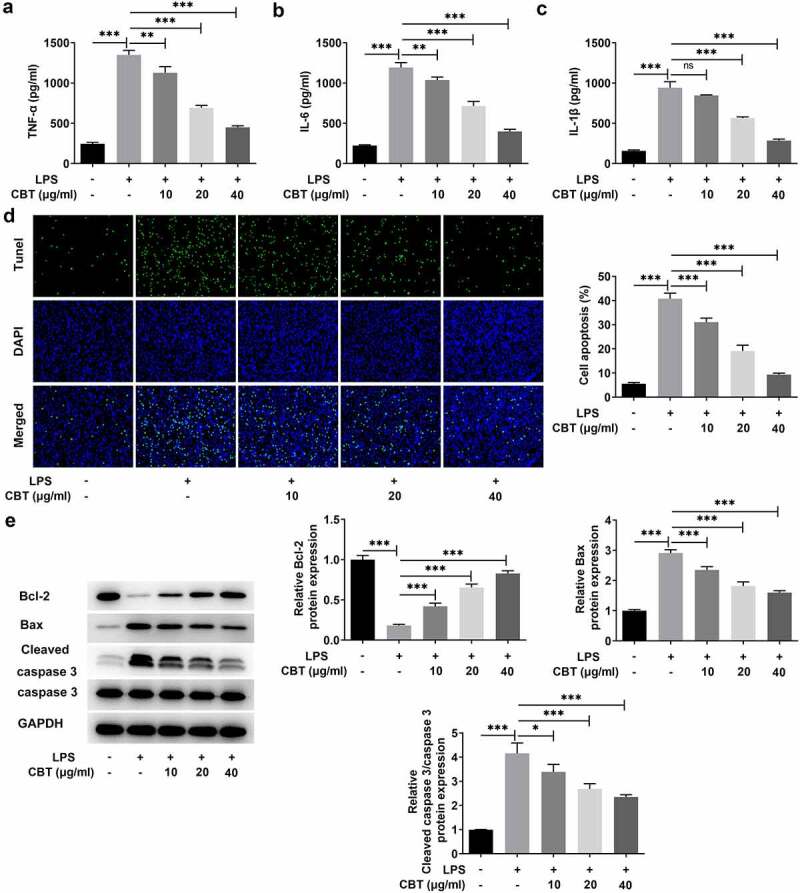
CBT ameliorates LPS-induced inflammatory response and apoptosis in ATDC5 cells.

It is well known that inflammation is a key factor in OA pathogenesis [21,22]. Therefore, suppression of inflammation has been proposed as a strategy for delaying the progression of OA. To elucidate the anti-inflammatory impacts of CBT on ATDC5 cells, the levels of inflammatory cytokines in LPS-induced ATDC5 cells were examined employing ELISA. It was apparently observed in Figure 2(a–c) that there was a sharp increase in the levels of inflammatory cytokines TNF-α, IL-6, IL-1β in the LPS group (vs Control) and a gradual decline in the levels of TNF-α, IL-6, IL-1β in the LPS+CBT (10, 20 and 40 μg/ml) groups. Additionally, more TUNEL-positive cells in the LPS group compared with the control group and fewer TUNEL-positive cells in the LPS+CBT groups were found in Figure 2(d). Accordingly, LPS induced a higher level of apoptosis, which was gradually reduced after CBT treatment at the concentration of 10, 20 and 40 μg/ml. Moreover, Figure 2(e) shows that there was a decrease in anti-apoptotic protein Bcl-2 and an increase in pro-apoptotic proteins Bax and cleaved caspase 3 in the LPS group (vs Control), as well as an increase in anti-apoptotic protein Bcl-2 and a decrease in pro-apoptotic proteins Bax and cleaved caspase 3 in the LPS+CBT groups. Overall, these results indicate that CBT concentration-dependently ameliorates LPS-induced inflammatory response and apoptosis in ATDC5 cells.

(a–c) The levels of TNF-α, IL-6 and IL-1β in LPS-induced ATDC5 cells treated with CBT (10, 20 and 40 μg/ml) were determined by means of ELISA. (d) The apoptosis rate of LPS-induced ATDC5 cells treated with CBT (10, 20 and 40 μg/ml) was assessed with the adoption of TUNEL. (e) The levels of Bcl-2, Bax and cleaved caspase 3 in LPS-induced ATDC5 cells treated with CBT (10, 20 and 40 μg/ml) were tested by the use of Western blot. *P < 0.05, **P < 0.01, ***P < 0.001.

### CBT promotes autophagy in LPS-induced ATDC5 cells

**Figure 3.**
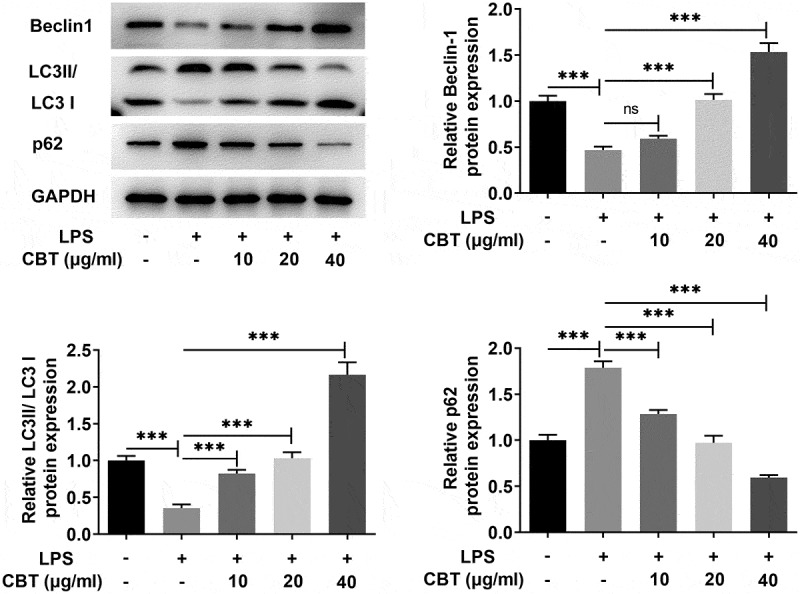
CBT promotes autophagy in LPS-induced ATDC5 cells.

Accumulating study confirms that activation of chondrocyte autophagy can effectively improve OA [23,24]. The objective of the experiments in this section sought to understand the effect of CBT on the autophagy of LPS-induced ATDC5 cells. Figure 3 clearly demonstrated the changes in expressions of autophagy-related proteins Beclin1, LC3II/LC3I and p62. The expressions of Beclin1 and LC3II/LC3I dropped by about half in the LPS group (vs Control), but gradually rose in a concentration-dependent manner in the LPS+CBT groups. The expression of p62 in each group was completely opposite to those of Beclin1 and LC3II/LC3I. These results provide important insights into the ability of CBT to promote the autophagy in LPS-induced ATDC5 cells.

The protein levels of Beclin1, LC3II/LC3I and p62 related to autophagy were detected by Western blot in LPS-induced ATDC5 cells treated with CBT (10, 20 and 40 μg/ml). ***P < 0.001.

### CBT inhibits SGK1 expression in LPS-induced ATDC5 cells

**Figure 4.**
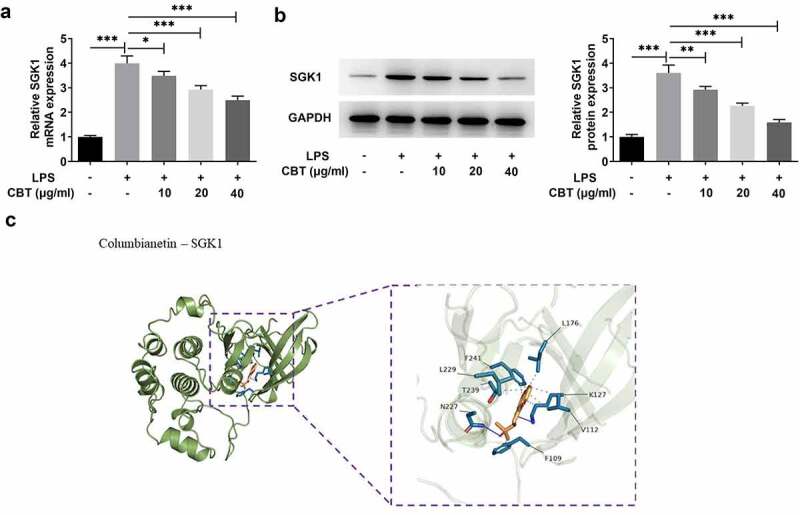
CBT inhibits SGK1 expression.

To explore the potential mechanisms of CBT on the regulation of LPS-induced chondrocyte damage, the interaction of CBT and SGK1 was predicted by molecular docking method. The structures of CBT and SGK1 were obtained from the PDB database (https://www.rcsb.org/) and constructed by the Molecular Operating Environment software. As indicated in Figure 4(a,b), the expression of SGK1 was sharply elevated in LPS-induced ATDC5 cells compared to the control group, but decreased steadily after the treatment of CBT (10, 20 and 40 μg/ml). SGK1 expression was minimized at the CBT concentration of 40 μg/ml. Therefore, CBT at a concentration of 40 μg/ml was chosen as the next experimental condition. Subsequently, the assay of molecular docking revealed that CBT can be mutually identified with SGK1 by spatial and ability matching (Figure 4(c)). Taken together, these results suggest that there is an association between CBT and SGK1, and CBT could inhibit SGK1 expression.

(a,b) SGK1 expression in LPS-induced ATDC5 cells treated with CBT (10, 20 and 40 μg/ml) was examined with the application of RT-qPCR and Western blot. (c) The relationship of CBT and SGK1 was verified by Molecular Docking. *P < 0.05, **P < 0.01, ***P < 0.001.

### CBT improves cell viability by inhibiting SGK1 and activating chondrocyte autophagy in LPS-induced ATDC5 cells

**Figure 5.**
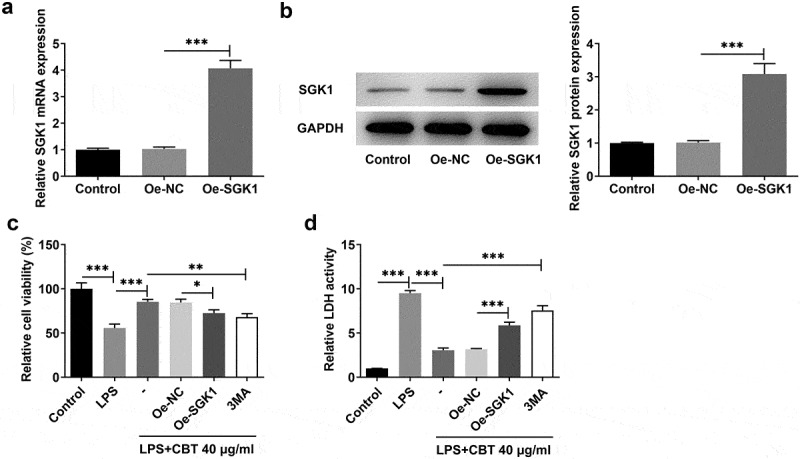
CBT improves cell viability by inhibiting SGK1 and activating chondrocyte autophagy in LPS-induced ATDC5 cells.

To understand whether CBT acts by binding SGK1 in LPS-induced ATDC5 cells, we transfected SGK1 overexpression plasmids into ATDC5 cells. Figure 5(a,b) showed that ATDC5 cells transfected with Oe-SGK1 showed a high expression of SGK1. Afterward, the autophagy inhibitor 3 MA (10 mM) was adopted to treat ATDC5 cells for 24 h to observe whether the effect of CBT on cell viability was mediated by autophagic pathway. It can be seen in Figure 5(c) that cell viability was clearly decreased in both LPS+CBT 40 μg/ml+Oe-SGK1 and LPS+CBT 40 μg/ml+3 MA groups. However, LDH activity were notably elevated in both groups (Figure 5(d)). The evidence presented in this section suggests that CBT could promote cell viability by inhibiting SGK1 and activating chondrocyte autophagy in LPS-induced ATDC5 cells.

(a,b) SGK1 expression in ATDC5 cells transfected with Oe-SGK1 was detected employing RT-qPCR and Western blot. (c) Cell viability of LPS-induced ATDC5 cells treated with Oe-SGK1 or 3 MA was tested adopting CCK-8. (d) LDH release level in ATDC5 cells treated with Oe-SGK1 or 3 MA was examined with the use of LDH kit. *P < 0.05, **P < 0.01, ***P < 0.001.

### CBT ameliorates LPS-induced inflammation and apoptosis by inhibiting SGK1 and activating chondrocyte autophagy in ATDC5 cells

**Figure 6.**
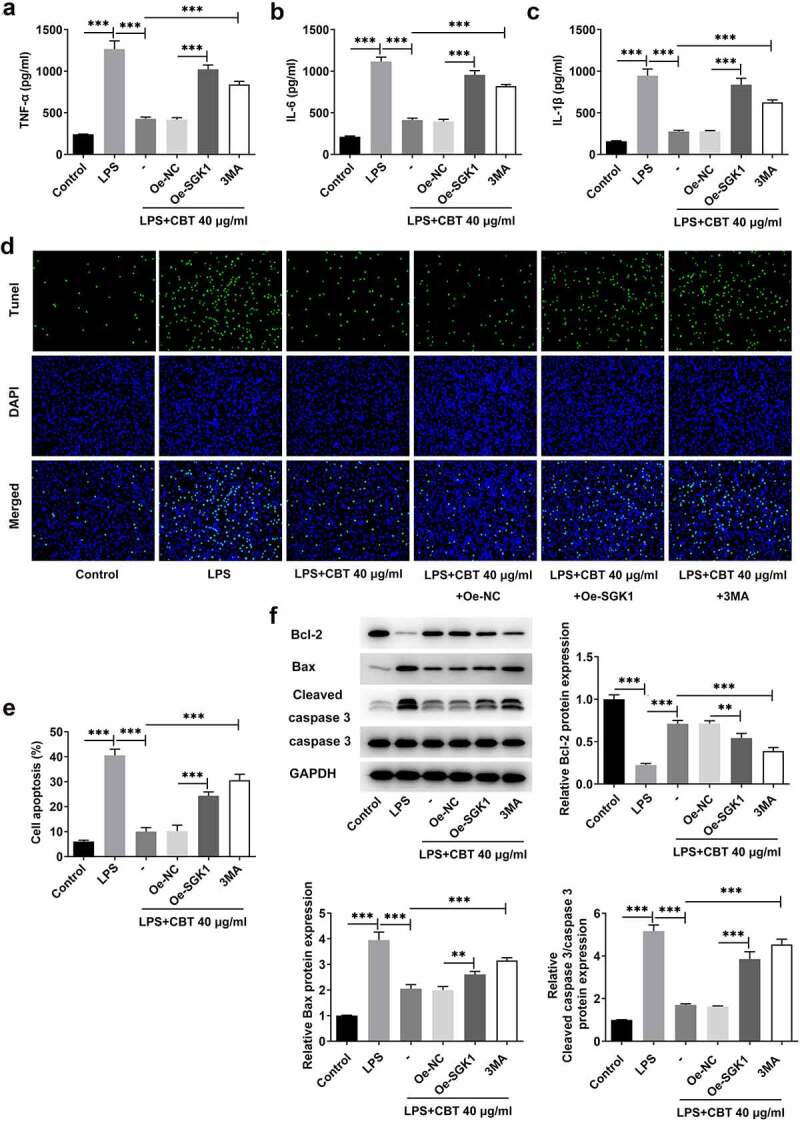
CBT ameliorates LPS-induced inflammation and apoptosis by inhibiting SGK1 and activating chondrocyte autophagy in ATDC5 cells.

The subsequent experiments were to determine whether CBT protected against LPS-induced inflammation and apoptosis in ATDC5 cells by inhibiting SGK1 and activating chondrocyte autophagy. It was found in Figure 6(a–c) that both Oe-SGK1 and 3 MA increased the levels of inflammatory cytokines TNF-α, IL-6 and IL-1β. Meanwhile, more TUNEL-positive cells and higher apoptosis rate calculated accordingly were found in the Oe-SGK1 and 3 MA groups (Figure 6(d,e)). More importantly, decreased expression of Bcl-2 and slowly increased expression of Bax and cleaved caspase 3 were also observed in LPS-induced ATDC5 cells treated with Oe-SGK1 or 3 MA, respectively (Figure 6(f)). Together, these results support the fact that CBT could also ameliorate LPS-induced inflammation and apoptosis by inhibiting SGK1 and activating chondrocyte autophagy in ATDC5 cells.

(a–c) The levels of TNF-α, IL-6 and IL-1β in CBT and LPS-stimulated ATDC5 cells treated with Oe-SGK1 or 3 MA were determined by means of ELISA. (d-e) The apoptosis rate of CBT and LPS-stimulated ATDC5 cells treated with Oe-SGK1 or 3 MA was assessed with the adoption of TUNEL. (f) The levels of Bcl-2, Bax and cleaved caspase 3 in CBT and LPS-stimulated ATDC5 cells treated with Oe-SGK1 or 3 MA were tested by the use of Western blot. ***P < 0.001.

## Discussion

OA seriously puts the health of middle-aged and elderly people at risk and imposes a heavy financial burden on patients [25]. However, the current treatment for OA is not ideal. The process of OA development is accompanied by inflammation and chondrocyte apoptosis in the bone and joint tissue [26]. Nevertheless, activation of cell autophagy may in turn protect chondrocytes from the negative effects of OA [27]. As a result, this study looked at the effects of CBT on the inflammation and apoptosis through activation of cell autophagy in ATDC5 cells and clarified its contribution to the therapeutic approach to OA.

CBT is a major active compound extracted from radix angelicae pubescentis that exerts anti-inflammatory effects in OA [10,28]. And its related compound Columbianetin acetate also clearly exhibited anti-inflammatory and analgesic activity [29]. This evidence allowed us to explore the possible anti-inflammatory function of CBT in OA. We first tested whether CBT was toxic to ATDC5 cells. According to the experimental results, different concentrations of CBT had little effect on the viability of ATDC5 cells. In addition, LPS is considered a key pro-inflammatory factor associated with OA pathogenesis [30]. Therefore, we established an *in vitro* cell model of OA using LPS and found a rapid decrease in viability of ATDC5 cells. A previous study has shown that CBT inhibits the proliferation of human melanoma cells [31]. Subsequently, we found that the viability of ATDC5 cells induced by LPS was enhanced after treatment with different concentrations of CBT. This illustrates that CBT protects ATDC5 cells from LPS-induced damage.

LPS is known to induce inflammatory responses in various diseases accompanied by elevated levels of pro-inflammatory cytokines such as TNF-α, IL-6 and IL-1β [32]. Consistently, TNF-α, IL-6, and IL-1β levels were also clearly increased in our study. Previous studies have shown that inhibition of these pro-inflammatory cytokines is effective in reducing inflammation in disease [33,34]. Additionally, CBT plays anti-inflammatory impacts in RAW 264.7 cells induced by LPS through inhibition of NO production [35]. Our results showed that inflammatory factors were reduced in CBT-treated ATDC5 cells, consistent with previous reports on the anti-inflammatory effects of CBT.

Apoptosis is one of the potential pathogenic mechanisms of OA, and the proportion of apoptotic chondrocytes in OA chondrocytes is higher than in normal tissues [36]. Thus, inhibition of chondrocyte apoptosis is an essential strategy for OA treatment. It is well documented that LPS can induce apoptosis in chondrocytes [37,38]. Therefore, in our experiments, we found a dramatic increase in the number of apoptotic ATDC5 cells under LPS induction. The anti-inflammatory effect of CBT on ATDC5 cells has been previously demonstrated. These clues suggest that CBT may also pose inhibitory effect on the apoptosis of chondrocyte. Our experiments also revealed that CBT ameliorated LPS-induced apoptosis of ATDC5 cells.

Autophagy can perform an important role in regulating and maintaining the metabolism of body capacity by degrading damaged cells [39,40]. Enhanced autophagy of chondrocytes prevents the progression of OA in articular cartilage [41]. However, LPS could inhibit cell autophagy in aerobic conditions [42]. This corresponded to the decreased expression of autophagy-related proteins Beclin1 and LC3II/LC3I in our experiments in ATDC5 cells induced by LPS. In addition, we also discussed the effect of CBT on cellular autophagy and found that CBT elevated the expression levels of Beclin1 and LC3II/LC3I. Beclin1 is a key molecule regulator of autophagy [43]. LC3II will be increased when autophagy is activated [44]. Additionally, it was found in our study that the level of p62 was elevated by LPS but decreased after treatment of CBT. This is because the autophagy adaptor protein p62 accumulates in response to impaired autophagy [45]. Therefore, our findings further supported the activating effect of CBT on the autophagy of ATDC5 cells induced by LPS.

SGK1 is a serine/threonine kinase, and its high expression is closely associated with the development of tumor [46]. Research noted that SGK1 was highly expressed in diverse cell types after inflammatory cytokine IL-6 stimulation [47]. Our study found that SGK1 expression was increased in the LPS group and decreased in the CBT group, suggesting an inhibitory effect of CBT on SGK1. Furthermore, molecular docking analysis also confirmed the targeted inhibition of CBT with SGK1. To further confirm that CBT may exert a protective effect on OA by inhibiting SGK1 and activating cellular autophagy, we constructed an SGK1 overexpression vector and blocked the autophagic pathway with the autophagy inhibitor 3 MA, and then tested whether CBT continued to affect OA. The results showed that CBT did not produce further protective effects against LPS-induced loss of cell viability, inflammation and apoptosis after SGK1 overexpression or autophagic pathway was blocked. This is because autophagy is involved in the process of OA by mediating apoptosis and the production of ROS [48]. However, blockage of the autophagic pathway instead promotes apoptosis and exacerbates the development of OA. However, data about the effects of CBT on the OA animal model has not been exhibited in this study, which is a limitation of the present study. Therefore, comprehensive analysis is required in the future to support the current conclusion, which will have great significance in the development of new therapeutic approaches.

## Conclusion

All of the studies discussed here support the hypothesis that CBT effectively attenuated LPS-induced inflammation and apoptosis, at least in part, by inhibiting SGK1 and activating autophagy. CBT may thus be useful as a potential therapeutic agent for OA.

## Data Availability

All experimental data in this study can be obtained by contacting the corresponding author.
